# Color fundus photograph-based diabetic retinopathy grading via label relaxed collaborative learning on deep features and radiomics features

**DOI:** 10.3389/fcell.2024.1513971

**Published:** 2025-01-09

**Authors:** Chao Zhang, Guanglei Sheng, Jie Su, Lian Duan

**Affiliations:** ^1^ School of Information Engineering, Suqian University, Suqian, Jiangsu, China; ^2^ School of Computer Science and Engineering, Xi’an University of Technology, Xi’an, China; ^3^ Department of Medical Informatics, Nantong University, Nantong, Jiangsu, China

**Keywords:** diabetic retinopathy grading, collaborative learning, radiomic features, highlevel deep features, label relaxation

## Abstract

**Introduction:**

Diabetic retinopathy (DR) has long been recognized as a common complication of diabetes, making accurate automated grading of its severity essential. Color fundus photographs play a crucial role in the grading of DR. With the advancement of artificial intelligence technologies, numerous researchers have conducted studies on DR grading based on deep features and radiomic features extracted from color fundus photographs.

**Method:**

We combine deep features and radiomic features to design a feature fusion algorithm. First, we utilize convolutional neural networks to extract deep features from color fundus photographs and employ radiomic methodologies to extract radiomic features. Subsequently, we design a label relaxation-based collaborative learning algorithm for feature fusion.

**Results:**

We validate the effectiveness of the proposed method on two fundus image datasets: the DR1 Dataset and the MESSIDOR Dataset. The proposed method achieved 96.86 of AUC on DR1 and 96.34 of AUC on MESSIDOR, which are better than state-of-the-art methods. Also, the divergence between the training AUC and testing AUC increases substantially after the removal of manifold regularization.

**Conclusion:**

Label relaxation can enhance the distinguishability of training samples in the label space, thereby improving the model's classification accuracy. Additionally, graph constraints based on manifold learning methods can mitigate overfitting caused by label relaxation.

## 1 Introduction

**FIGURE 1:**
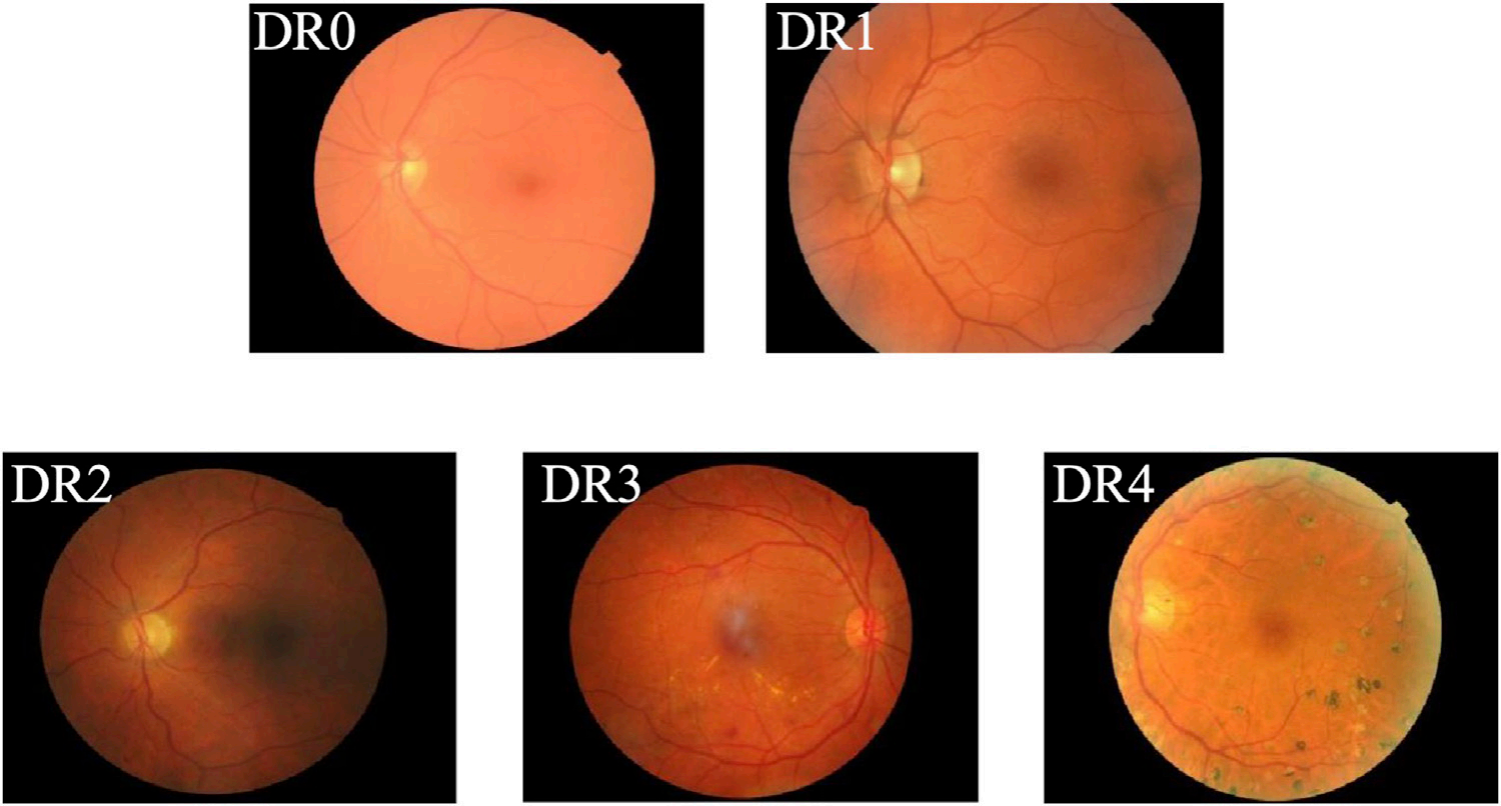
Example of DR grading.

Diabetes Mellitus (DM) is a chronic metabolic disorder typically characterized by insufficient insulin production or ineffective insulin action, resulting in elevated blood glucose levels and a cascade of severe complications (Nițulescu et al., 2023; Zhang et al., 2022). Diabetic Retinopathy (DR) has long been recognized as a prevalent complication of diabetes and is the primary cause of vision impairment among diabetic patients, with severe cases potentially leading to permanent blindness (Tan and Wong, 2023). In recent years, the prevalence and associated blindness rates of DR have risen rapidly. According to the World Health Organization (WHO), the number of individuals affected by DR is expected to reach 552 million by 2030, making it a leading cause of blindness among the working-age population (Wang et al., 2022). In clinical practice, early-stage DR often presents without noticeable visual symptoms, making it difficult to detect. However, once visual impairment occurs, it results in irreversible damage for the patient. According to the International Clinical Diabetic Retinopathy Disease Severity Scale (Dai et al., 2024), the progression of DR is categorized into five stages: DR0 to DR4, with higher stages indicating more severe disease, as shown in Figure 1. Specifically, DR0 indicates the absence of apparent lesions; DR1, DR2, and DR3 represent mild, moderate, and severe non-proliferative diabetic retinopathy (NPDR), respectively. DR4 is classified as proliferative diabetic retinopathy (PDR), the most severe stage, during which patients commonly experience acute vision deterioration, potentially leading to complete blindness. Therefore, regular retinal screening is essential for diabetic patients, facilitating the early detection and accurate grading of DR. Different treatment approaches can then be implemented based on the severity of the condition, promoting early identification, timely intervention, and effective treatment to prevent vision loss. Thus, establishing an AI-aided diagnostic model to assist in the diagnosis of DR is both a feasible and necessary solution. AI-aided diagnostic models can alleviate the workload of specialized physicians (Wu et al., 2024), significantly enhance the efficiency of DR screening, and provide a second, objective opinion during the diagnostic process. This approach reduces the subjectivity inherent in human assessments, enabling more accurate diagnoses and timely treatment for DR patients, ultimately lowering the risk of vision loss due to the disease.

The aim of developing an AI-assisted diagnostic model is to enable precise automated grading of diabetic retinopathy severity. Traditional methods for DR grading require the design of hand-crafted feature extraction algorithms, alongside the use of general classifiers such as Support Vector Machines (SVM) or Random Forests (RF) and their various adaptations to classify the severity of DR (Nayak et al., 2008; De la Calleja et al., 2014; Carrera-Escalé et al., 2023; Soren et al., 2024). For instance, Nayak et al. (2008) utilized morphological operations and texture analysis to detect regions of hard exudates, vascular areas, and contrast features, subsequently inputting these characteristics into an Artificial Neural Network (ANN) for disease staging, achieving an accuracy of 93%. De la Calleja et al. (2014) applied Local Binary Pattern (LBP) algorithms to extract local features, which were then analyzed using ANN, SVM, and Random Forest (RF) classifiers for DR grading. Their results indicated that RF performed best within a dataset of 71 images, achieving an accuracy of 97.46%. In addition, radiomics has been demonstrated its power in DR grading, which aims to analyze a large number of quantitative features from medical imaging data to uncover potential biological information and clinical relevance. For instance, Laura et al. extracted radiomics features from angiography (OCT) images and established machine learning models to classify DM, DR and referable-DR (R-DR) (Carrera-Escalé et al., 2023). Soren et al. constructed a DR grading model and found that radiomics features are significantly different for increasing levels of DR severity (Soren et al., 2024). While these methods demonstrate significant potential, they tend to rely heavily on prior knowledge and still require improvements under complex imaging conditions. In recent years, deep learning algorithms have made significant advancements in the field of computer vision, with Convolutional Neural Networks (CNNs) emerging as the dominant architecture for medical image analysis due to their powerful capabilities in high-level feature extraction and representation. Ting et al. (2017) from the National University of Singapore utilized the VGG network for DR screening. Similarly, the Krause research team at Google Research employed the Inception V4 model to automatically detect DR in color fundus photographs and predict its severity (Krause et al., 2018). However, given the complexities involved in the DR grading task, relying solely on CNN models has not yielded optimal results. Consequently, researchers have explored various methods to enhance model performance for DR classification. Sugeno et al. (2021) applied a Laplacian filter with a kernel size of 5 to filter out blurred images, calculating the standard deviation of the Laplacian operator’s output to eliminate noise. The resulting clear color fundus photographs were then input into a CNN for DR prediction, significantly improving model performance compared to previous approaches. Bellemo et al. (2019) utilized two different deep learning models, VGG and ResNet, to extract features and employed ensemble learning to integrate the prediction scores from both models, thereby achieving more accurate results. Tariq et al. (2021) adopted deep transfer learning, combining the classification results from various CNNs to diagnose DR effectively.

From above-mentioned studies, we can see that both hand-crafted features and deep features play significant roles in AI-aided DR grading. However, few studies focus on how to mine the complementary or consistent patterns from them to improve DR grading performance. In multi-view learning, it has been demonstrated that mining complementary or consistent patterns from different views can improve the classification performance. Therefore, in this study, we utilize convolutional neural networks to extract high-level deep features from color fundus photographs and employ radiomic methodologies to extract radiomic features. Then we design a label relaxation-based collaborative learning algorithm for high-level deep feature and radiomic feature fusion. That is to say, high-level deep features can be considered as one view, radiomic features can be considered as another view. The method aims to mine the complementary or consistent patterns from the two views.

The rest sections are organized as follows. Section 2 gives data preprocessing steps. In Section 3, we show our method. In Section 4, we report our experimental results from different aspects. In the final section, we conclude this study.

## 2 Data preprocessing

In this study, we collect two public fundus image datasets for our experimental studies. Based on the two public datasets, we construct a binary classification task which aims to distinguish abnormal and normal color fundus photographs. In the following, we briefly introduce the two datasets and present the data preprocessing steps.

### 2.1 Dataset

#### 2.1.1 DR1

DR1 dataset is provided by the Ophthalmology Department at the Federal University. It comprises 1,014 color fundus photographs, in which 687 images are normal and 327 iamges are abnormal. Among the abnormal images, 245 exhibit bright lesions, while 191 display red lesions. Additionally, 109 images show evidence of both bright and red lesions (Rocha et al., 2012). The images were captured using a Topcon TRC-50X mydriatic camera, all with a resolution of 640 × 480 pixels. Each image has been manually annotated by three medical experts to indicate the presence or absence of bright or red lesions. According to the evaluators, normal images show no signs of Diabetic Retinopathy (DR), while abnormal images may present various lesions, including exudates, hemorrhages, and microaneurysms.

#### 2.1.2 MESSIDOR

MESSIDOR is another available fundus image dataset (Decencière et al., 2014). It comprises 1,200 eye color fundus photographs collected from three different sites, with 800 images obtained with pupil dilation and 400 without. The images were captured using a color video 3 CCD camera at resolutions of 1440 × 960, 2240 × 1488, or 2304 × 1536 pixels. Based on the severity classification of DR in patients, the dataset is divided into five levels. Specifically, MESSIDOR includes 546 images classified as DR0 (normal), 153 images as DR1 (mild), 247 images as DR2 (moderate), and 254 images as DR3 (severe), with level 3 encompassing both severe non-proliferative retinopathy and proliferative retinopathy.

### 2.2 Preprocessing

In this study, we aim to extract radiomic features and high-level deep features from the two datasets. For radiomic feature extraction, according to Liang’s suggestion (Liang et al., 2021), all color fundus photograph are cropped into a fixed resolution of 350 × 350 pixels, and the green channel is extracted. Additionally, as suggested by Liang (Liang et al., 2021), Contrast Limited Adaptive Histogram Equalization (CLAHE) (Zimmerman et al., 1989) is employed to mitigate the influence of external factors as much as possible. For high-level deep feature extraction, all color fundus photographs are cropped into a fixed resolution of 224 × 224 pixels.

## 3 Methodology

**FIGURE 2:**
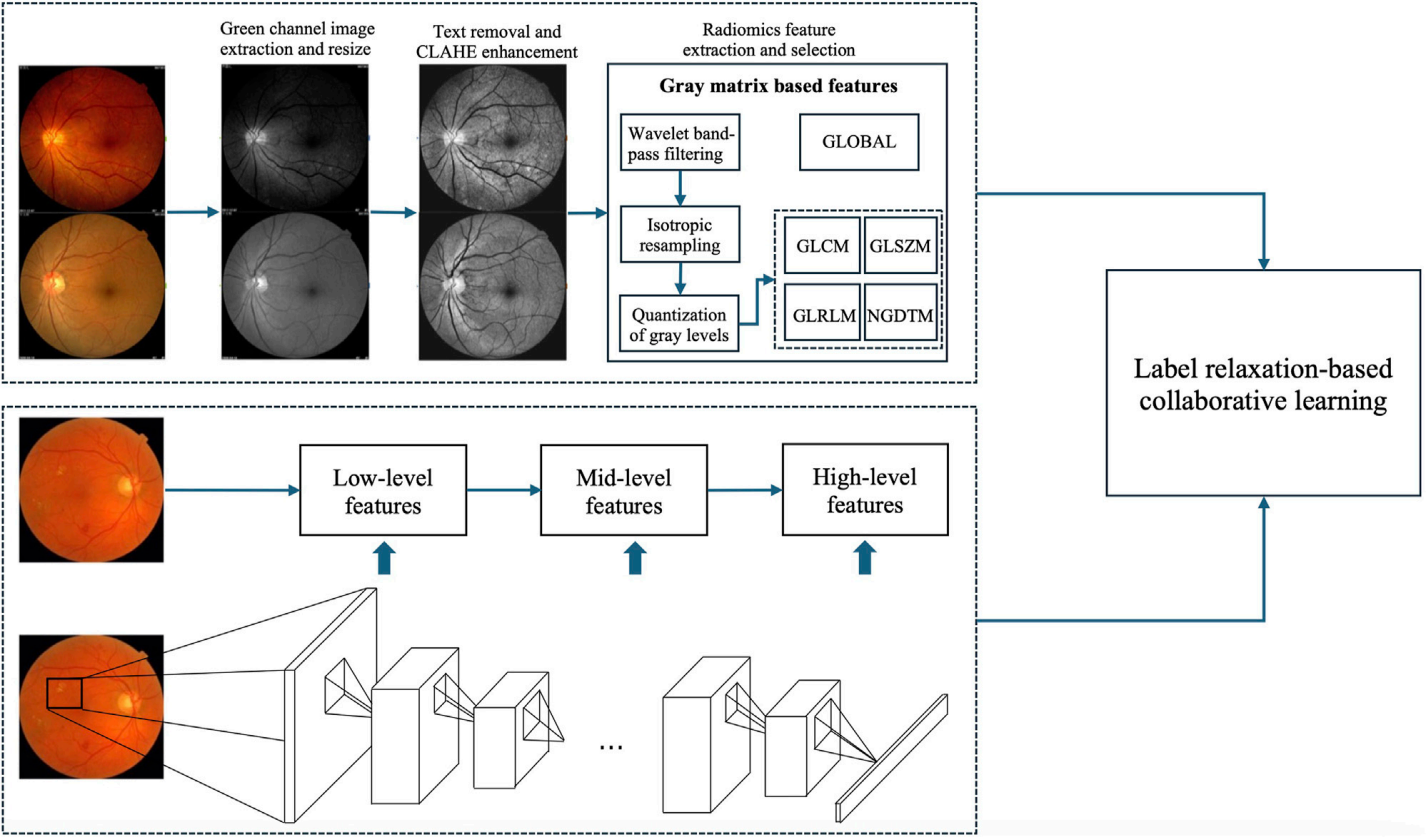
Framework of the proposed method.

Figure 2 shows the framework of the proposed method, which contains three components, radiomics feature extraction, high-level deep feature extraction and label relaxation-based collaborative learning.

### 3.1 Radiomic feature extraction

Radiomic features capture both the homogeneity present in the image and the structural arrangement of the object’s surface, which may exhibit gradual or periodic variations. To extract a richer set of radiomic features from the regions of interest (ROIs), we evaluated various types of texture features. Unlike previous radiomics studies, our approach did not require manual delineation of any ROIs. Instead, we employed a threshold-based segmentation method to generate the corresponding mask automatically.

Global features are typically first-order statistical attributes that capture the statistical characteristics of images. Four common matrix-based texture features used in texture classification include the Gray Level Co-occurrence Matrix (GLCM), the Gray Level Run Length Matrix (GLRLM), the Gray Level Size Zone Matrix (GLSZM), and the Neighborhood Gray Tone Difference Matrix (NGTDM).

The GLCM represents the joint distribution of two pixels with a specific spatial relationship, effectively functioning as a joint histogram of pixel gray value pairs, thereby providing second-order statistics. The GLRLM captures comprehensive information about the gray images, reflecting variations in direction, adjacency, and amplitude. The GLSZM calculates the number of connected voxels in an image. Adjacent voxels sharing the same gray level are considered connected. NGTDM quantifies the difference between the gray value of a specific point and the average gray value of its surrounding neighborhood, storing the cumulative differences between all gray levels and their average gray values within the matrix.

Prior to extracting these features, we conducted three preprocessing operations on the images: WBPF (wavelet band-pass filtering), IR (isotropic resampling), and GCLT (quantization and gray level transformation), as suggested by Vallières (Vallières et al., 2015), to enhance the richness of the extracted texture features.

#### 3.1.1 WBPF

To mitigate the influence of noise and enhance the differentiation among various bandwidths, we employed the “Sym8” wavelet basis function for the decomposition and reconstruction of images in this study. The ratio of high and low-frequency coefficients is denoted by “W”.

#### 3.1.2 IR

To enrich the extracted texture features, this operation was performed to obtain images at varying resolutions. The size of the isotropic resampling is represented by “S”.

#### 3.1.3 GCLT

To reduce time complexity and facilitate the extraction of additional texture features, this operation converts images to different gray levels. Two key parameters in this process are the quantization algorithm and the number of gray levels, denoted as ‘Algo’ and ‘Ng’, respectively. In this work, we selected two quantization algorithms: equal-probability (Vallières et al., 2015) and Lloyd-Max quantization (Max, 1960). Table 1 shows the number of extracted radiomics features.

**TABLE 1:** Categories of radiomics features and corresponding number of features in each category.

Categories of radiomics features	Number of features	Category description
GLCM	10	Gray Level Co-occurrence Matrix
GLRLM	15	Gray Level Run Length Matrix
GLSZM	15	Gray Level Size Zone Matrix
NGDTM	7	Neighborhood Gray Tone Difference Matrix
LBP	256	Local binary patterns
MRELBP	900	Median Robust Extended Local Binary Pattern
BPPC	1,087	Binary Pattern of Phase Congruency
IWBC	2048	Improved Weber Local Descriptor
GDP	256	Gradient directional pattern
LFD	512	Local Frequency Descriptor
MBC	3,090	Monastic Binary Coding
LTrP	256	Transition of intensity change in different directions over a local area
GLOBAL	3	Frst-order statistical features

### 3.2 High-level deep feature extraction

Convolutional Neural Networks (CNNs) comprise multiple layers, enabling the learning of data representations at various levels of abstraction. The representation generated by each layer is derived from that of the preceding layer. CNNs can be trained using an end-to-end BP algorithm, as this approach effectively integrates feature extraction with classification processes. Generally, convolutional layers are usually taken as feature extractors, while fully connected layers are taken as classifiers. The lower layers of a CNN capture low-level features, whereas the higher layers identify high-level features that can describe either the entirety or specific components of objects within images. Current studies indicate that features extracted from pre-trained CNNs are highly powerful for a range of classification tasks (Alghamdi et al., 2024; Hakim et al., 2023; Lankasena et al., 2024). In this study, we utilize pre-trained AlexNet provided by the Matlab toolbox “MatConvNet” as feature extractors to extract high-level deep features, as illustrated in Figure 2.

### 3.3 Label relaxation-based collaborative learning (LRCL)

To effectively fuse radiomic features and high-level deep features, we propose a novel label relaxation-based collaborative learning model termed as LRCL. Some main mathematical notations used in the study are summarized in Table 2.

**TABLE 2:** Main mathematical notations.

Notations	Description
$X_{k}$	Dataset of the k-th view
Y	Label matrix
H	Nonnegative label relaxation matrix
U	Luxury matrix
$O_{k}$	Edge weight matrix of the k-th view
$L_{k}$	Laplacian matrix of the k-th view
K	Number of views
N	Number of samples
$\omega_{k}$	Weight of the k-th view

**FIGURE 3:**
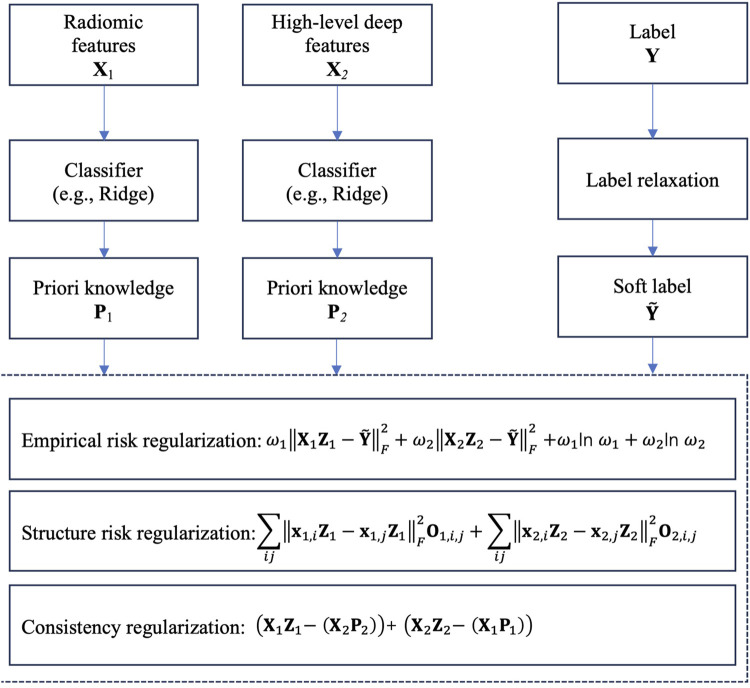
Core idea of LRCL.

The core idea of LRCL is illustrated in Figure 3. From Figure 3, we see that two regularizations are used to reach the goal of collaborative learning. The first one is view-weighting. Specifically, “Shannon entropy” is used to automatically learn the weight $\omega_{1}$ of radiomic features and the weight $\omega_{2}$ of high-level deep features. The view weights are then embedded into empirical risk calculation. The second one is the consistency regularization which is used to mine the consistent patterns across radiomic features and high-level deep features. Specifically, we firstly train a classifier, e.g., Ridge on the radiomic feature space and the high-level deep feature space, respectively, then the trained parameter on each feature space, e.g., the transformation matrix in Ridge is taken as priori knowledge for the following consistent pattern mining. Then in the stage of consistent pattern mining, we construct a consistency regularization, as shown in Figure 3, to keep the consistency across the two feature space.

Moreover, to future improve the performance of collaborative learning, we introduce a label relaxation technique to re-construct the label space. Suppose that Y is the original label matrix, in which the element $Y_{ij}=1$ means the *i*-th sample belonging to the *j*-th class, $Y_{ij}=0$ means the *i*-th sample belonging to other classes. Therefore, Y is a strict binary matrix. To relax the strict binary matrix, we introduce a luxury matrix U , which is defined in (Equation 1).

$$U_{ij}=\left\{\begin{array}{c} +1\;if\;Y_{ij}=1\\ -1\;if\;Y_{ij}=0 \end{array}\right.$$ (1)

With U **,** the original label matrix Y can be relaxed to $\tilde{Y}$ by the following (Equation 2).

$$\tilde{Y}=Y+U⊙H$$ (2)

where **H** is an nonnegative label relaxation matrix needed to be learned on training samples, and ⊙ is a Hadamard product operator.

In previous studies (Wang et al., 2021; Fang et al., 2017), some authors indicated that although label relaxation can enlarge the class margins between different classes and allows the classifier to have greater flexibility in fitting the labels, it may increase the risk of overfitting. To reduce overfitting risk, inspired by manifold learning, we suppose that if two samples on the radiomic feature space or the high-level feature space are in a same manifold, then in the relaxed label space, the two samples should be kept as close as possible. To achieve this goal, on each feature space, we construct a undirected graph and define the edge weight $O_{k,ij}$ as follows.

$$O_{k,ij}=\left\{\begin{array}{c} e^{-\frac{{\left\|x_{k,i}-x_{k,j}\right\|}_{F}^{2}}{\delta }}\\ 0\;otherwise \end{array}\right.\;if\;x_{k,i}\;and\;x_{k,j}\;are\;in\;the\;same\;manifold$$ (3)

In (Equation 3), δ is a user-defined kernel width. It can be seen from Equation 3 that in the *k*-th feature space, if two samples $x_{k,i}\;and\;x_{k,j}$ have the same label in the feature space, the closer their distance, the large weight $W_{k,ij}$ will be. Therefore, by minimizing the following objective function shown in (Equation 4), we can keep $x_{k,i}\;and\;x_{k,j}$ close in the relaxed label space.

$$\sum_{k=1}^{K}\sum_{ij}^{N}{\left\|x_{k,i}Z_{k}-x_{k,j}Z_{k}\right\|}_{F}^{2}O_{k,ij}=tr\left(Z_{k}^{T}X_{k}^{T}L_{k}X_{k}Z_{k}\right),$$ (4)

where $x_{k,i}Z_{k}$ represents $x_{k,i}$ in the relaxed label space by $Z_{k}$ . $L_{k}$ is the Laplacian matrix of the k-th view.

Consequently, based on the analysis above, the final objective of LRCL can be expressed in (Equation 5).

$$J\left(Z_{k},H,\omega_{k}\right)=\sum_{k=1}^{K}\omega_{k}{\left\|X_{k}Z_{k}-\left(Y+U⊙H\right)\right\|}_{F}^{2}+\omega_{k}\ln\omega_{k}+\alpha tr\left(Z_{k}^{T}X_{k}^{T}L_{k}X_{k}Z_{k}\right)+\beta \left({\left\|X_{k}Z_{k}-\frac{1}{K-1}\sum_{t=1,t\neq k}^{K}X_{t}P_{t}\right\|}_{F}^{2}\right),s.t.\;H\geq 0,\sum_{k=1}^{K}\omega_{k}=1$$ (5)

where α and β are user-defined parameters for balance controlling.

By applying the Lagrangian multiplier optimization method, we can get the closed-form solutions of $Z_{k}$ **,** H and $\omega_{k}$ , as shown in (Equation 6, Equation 7 and Equation 8).

$$Z_{k}={\left[\left(\omega_{k}+\beta \omega_{k}\right)X_{k}^{T}X_{k}+\alpha X_{k}^{T}L_{k}X_{k}\right]}^{-1}\left(\omega_{k}X_{k}^{T}\left(Y+U⊙H\right)+\beta \omega_{k}X_{k}^{T}\frac{1}{K-1}\sum_{t=1,t\neq k}^{K}X_{t}P_{t}\right).$$ (6)

$$\omega_{k}=\frac{e^{-{\left\|X_{k}-\left(Y+U⊙H\right)\right\|}_{F}^{2}-\beta {\left\|X_{k}A_{k}-\left(\frac{1}{K-1}\sum_{t=1,t\neq k}^{K}X_{t}P_{t}\right)\right\|}_{F}^{2}}}{\sum_{t=1}^{K}e^{-{\left\|X_{t}-\left(Y+U⊙H\right)\right\|}_{F}^{2}-\beta {\left\|X_{t}A_{t}-\left(\frac{1}{K-1}\sum_{t=1,t\neq k}^{K}X_{t}P_{t}\right)\right\|}_{F}^{2}}}$$ (7)

$$H=\max\;\left(U⊙\left(\sum_{k=1}^{K}{\omega_{k}X}_{k}Z_{k}-Y\right),0\right)$$ (8)

With **H,** $\omega_{k}$ and $Z_{k}$ , the algorithm of **LRCL** can be specified as follows. The time complexity of LRCL is mainly contributed by the computation of Step 6, Step 7 and Step 8. It can be seen from (Equation 6) that the time complexity of computing $Z_{k}$ is $O\left(N^{3}\right)$ . It can be seen from (Equation 7) that the time complexity of computing $\omega_{k}$ is $O\left(N^{2}\right)$ . It can be seen from (Equation 8) that the complexity of computing H is $O\left(N^{2}\right)$ . Therefore, the time complexity of LRCL is $O\left(t\left(N^{3}+2N^{2}\right)\right)$ .

**Table:** 

Label relaxation-based collaborative learning (LRCL)
Input: $X_{k}$ , α , and β Output: **H,** $\omega_{k}$ and $Z_{k}$ Procedures: Step 1. Use a classifier, e.g., Ridge to compute priori knowledge $P_{k}$ Step 2. Compute the Laplacian matrix $L_{k}$ Step 3. Randomize **H** and $\omega_{k}$ under $H\geq 0,\sum_{k=1}^{K}\omega_{k}=1$ Step 4. Set *t*=0 **Repeat** Step 5. *t*←*t*+1 Step 6. Employ (Equation 6) to compute ${Z_{k}}^{\left(t\right)}$ Step 7. Employ (Equation 7) to compute ${\omega_{k}}^{\left(t\right)}$ Step 8. Employ (Equation 8) to compute $H^{\left(t\right)}$ **Until** $\left\|{Z_{k}}^{\left(t\right)}-{Z_{k}}^{\left(t-1\right)}\right\|<10^{-8}$

## 4 Experimental results

### 4.1 Settings

On each dataset, 60% samples are used for feature selection and classification model training. 20% samples are used for validation which aims to select best model. The rest 20% samples are used for model testing.

For deep feature extraction, fine-tuning is conducted over 30 epochs using stochastic gradient descent with minibatches of size 50. The learning rate begins at 0.1 and decreases linearly to 0.0001 over the course of these epochs. Weight decay and momentum are set at 0.0005 and 0.95, respectively. To mitigate overfitting, fine-tuning typically requires a moderately sized dataset. Given the limited size of our datasets, we employ label-preserving transformations to artificially augment the data (Dan et al., 2012). The data augmentation techniques utilized include vertical flipping, horizontal flipping, and rotation at various angles. This approach increases the dataset size by a factor of 15. For each fundus image dataset, the same data augmentation methods are applied to both the training and test sets.

The evaluation of the proposed method can be organized into two parts. In the first part, as shown in Section 4.2, we report the comparison results between the proposed method and state-of-the-art multi-view methods (Xie et al., 2023; Zhou et al., 2023; Luo et al., 2023; Xu et al., 2024; Zhang et al., 2020), and the comparison results between the proposed method and traditional machine learning methods. In the second part, as shown in Section 4.3, we carry out ablation studies by removing some core components to demonstrate the power of the removed components.

The parameters of state-of-the-art multi-view methods are set based on the authors’ suggestions. The parameters of traditional machine learning methods are set by cross-validation with grid search. Pearson Score and Fisher Score are employed as the feature selection methods. After feature selection, the final number of radiomics features is 30, the final number of deep features is 35.

SP (Specificity, %), SN (Sensitivity, %), ACC (Accuracy, %) and AUC (Area under the ROC curve, %) are employed to measure the performance of all methods.

### 4.2 Result analysis

Table 3 shows the comparison results between the proposed method and state-of-the-art multi-view methods. From Table 3, we can see that the proposed method performs better than state-of-the-art multi-view methods on both DR1 and MESSIDOR (best results are marked in bold). In this study, we adopt view-weighting and consistency regularizations to mine the consistent patterns across the radiomic feature space and the high-level deep feature space. In some of the state-of-the -art methods, only view-weighting are used for collaborative learning, which may ignore the hidden patterns across different views. To further demonstrate the promising performance of the proposed method, we compare the proposed method with some traditional machine learning methods, the comparison results are shown in Table 4. The radiomic feature and the high-level deep features are directly combined as a new feature vector, and is taken as the input of the traditional machine learning methods. From Table 4, we see that the proposed method performs the best. This is because direct feature combination cannot effectively provide classification patterns.

**TABLE 3:** Comparison results between the proposed method and state-of-the-art multi-view methods.

Model	DR1				MSESSIDOR			
	SP	SN	ACC	AUC	SP	SN	ACC	AUC
Xie et al. (2023)	93.78*	90.11*	91.17*	95.76	94.34	83.46*	90.55*	92.45*
Zhou et al. (2023)	96.87*	89.45*	92.67*	94.12*	94.32	82.53*	92.64*	92.65*
Luo et al. (2023)	95.27*	88.47*	90.90*	93.82*	91.18*	81.65*	91.45*	94.64*
Xu et al. (2024)	93.41*	87.67*	90.55*	95.67	93.76*	85.32*	92.08*	93.36*
Zhang et al. (2020)	93.34*	87.90*	92.09*	91.98*	94.11*	85.32*	91.87*	95.87
Proposed	**98.43**	**91.78**	**93.12**	**96.86**	**95.11**	**87.03**	**94.01**	**96.34**

*means the difference is at 5% significant level, i.e., *p*-value is less than 0.05. The best performance is marked in bold.

**TABLE 4:** Comparison results between the proposed method and traditional machine learning methods.

Model	DR1				MSESSIDOR			
	SP	SN	ACC	AUC	SP	SN	ACC	AUC
SVM	92.11*	88.76*	90.45*	94.53*	92.34*	80.11*	87.56*	90.11*
Ridge	93.12*	85.43*	90.34*	93.34*	92.45*	80.23*	90.34*	89.23*
XGBoost	93.21*	86.27*	87.34*	92.53*	90.13*	79.61*	89.23*	93.12*
DT	91.45*	85.13*	84.23*	92.45*	91.34*	82.34*	89.12*	90.21*
RF	90.76*	85.64*	90.43*	92.34*	92.19*	80.25*	90.12*	92.34*
Proposed	**98.43**	**91.78**	**93.12**	**96.86**	**95.11**	**87.03**	**94.01**	**96.34**

*means the difference is at 5% significant level, i.e., *p*-value is less than 0.05. The best performance is marked in bold.

### 4.3 Ablation study

**FIGURE 4:**
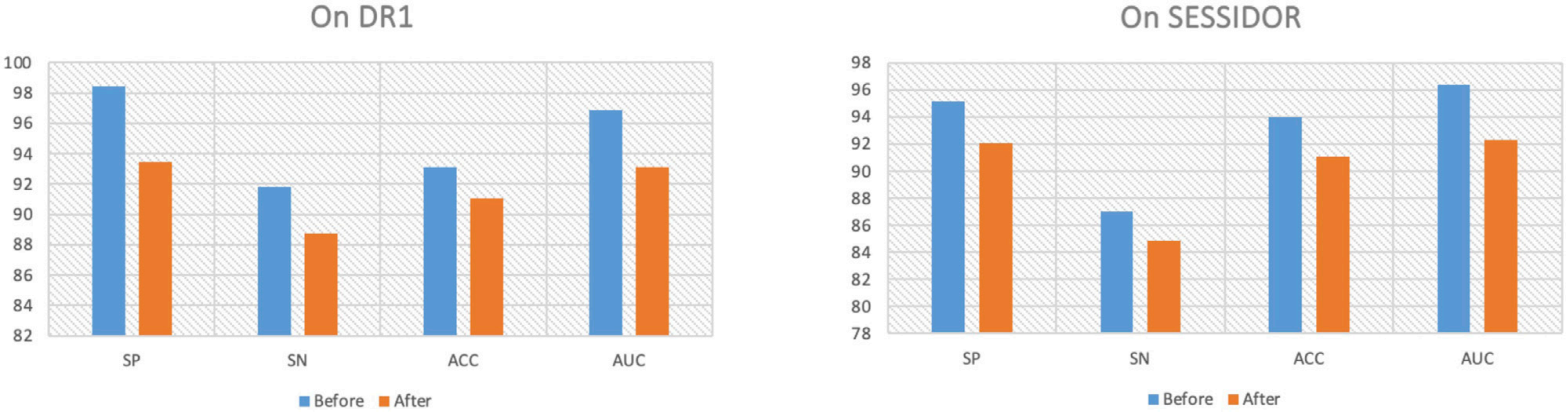
Ablation of consistency regularization.

**FIGURE 5:**
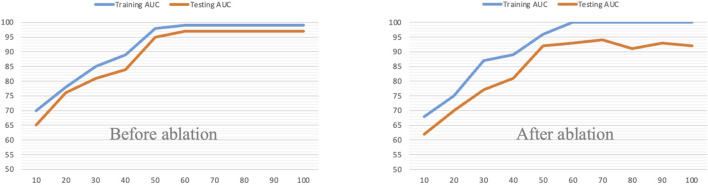
Ablation of manifold regularization.

To further demonstrate why the proposed method performs well, we first remove the consistency regularization from the objective function. Figure 4 shows the comparison results before/after consistency regularization ablation. From Figure 4, we see that when we remove the consistency regularization, the performance drops significantly, which indicates that the consistent patterns across the radiomic feature space and the high-level deep feature space can indeed improve the classification performance. Secondly, we remove the manifold regularization to observation model generalization. Figure 5 shows the training and testing AUC against iteration on DR1 before/after ablation. From Figure 5, we see that the divergence between the training AUC and testing AUC increases substantially after the removal of manifold regularization, which indicates a lower generalization ability when manifold regularization is removed, in comparison to the ablation preceding before.

## 5 Conclusion

In this study, we design a feature fusion framework to fuse high-level deep features and radiomic features for DR grading. Comparing with existing similar studies, the proposed framework has more freedom to fit the labels with low overfitting risk. In addition, comparing with direct feature combination, we design the consistency regularization to mine the consistent patterns across the high-level deep features and radiomic features, which can improve the DR grading performance. Experimental results demonstrate the effectiveness of the proposed framework. However, this study still has certain limitations. For instance, in addition to consistency patterns, different feature spaces may also exhibit complementary patterns, and effectively exploring these complementary patterns warrants further investigation.

## Data Availability

Publicly available datasets were analyzed in this study. This data can be found here: https://www.adcis.net/en/third-party/messidor/ and https://paperswithcode.com/dataset/retinal-lesions.
